# Generation of a Syngeneic Heterozygous *ACVRL1^(wt/mut)^* Knockout iPS Cell Line for the In Vitro Study of HHT2-Associated Angiogenesis

**DOI:** 10.3390/cells12121600

**Published:** 2023-06-10

**Authors:** Li Xiang-Tischhauser, Michael Bette, Johanna R. Rusche, Katrin Roth, Norio Kasahara, Boris A. Stuck, Udo Bakowsky, Maria Wartenberg, Heinrich Sauer, Urban W. Geisthoff, Robert Mandic

**Affiliations:** 1 VASCERN HHT Reference Centre, Department of Otorhinolaryngology, Head and Neck Surgery, University Hospital Marburg, Philipps-Universität Marburg, 35033 Marburg, Germany; 2 Department of Molecular Neuroscience, Institute of Anatomy and Cell Biology, Philipps-Universität Marburg, 35037 Marburg, Germany; 3 Cellular Imaging Core Facility, Center for Tumor Biology and Immunology (ZTI), Philipps-Universität Marburg, 35043 Marburg, Germany; 4 Department of Oral- and Cranio-Maxillofacial Surgery, University Hospital Marburg, Philipps-Universität Marburg, 35043 Marburg, Germany; 5 Department of Histology and Developmental Biology, Tokyo Dental College, Tokyo 101-0061, Japan; 6 Department of Pharmaceutics and Biopharmaceutics, Philipps-Universität Marburg, 35037 Marburg, Germany; 7 Department of Internal Medicine I, Division of Cardiology, University Hospital Jena, Friedrich Schiller University, 07747 Jena, Germany; 8 Department of Physiology, Justus-Liebig University Giessen, 35392 Giessen, Germany

**Keywords:** Morbus Osler, HHT2, ACVRL1, iPSC

## Abstract

Hereditary hemorrhagic telangiectasia (HHT) type 2 is an autosomal dominant disease in which one allele of the *ACVRL1* gene is mutated. Patients exhibit disturbances in TGF-beta/BMP-dependent angiogenesis and, clinically, often present with severe nosebleeds as well as a reduced quality of life. The aim of our study was to use CRISPR/Cas9 to knockout *ACVRL1* in normal induced pluripotent stem cells (iPSCs) and evaluate the effects on TGF-beta- and BMP-related gene expression as well as angiogenesis. The CRISPR/Cas9 knockout of the *ACVRL1* gene was carried out in previously characterized wild-type (*ACVRL1^wt/wt^*) iPSCs. An HHT type 2 iPS cell line was generated via a single-allele knockout (*ACVRL1^wt/mut^*) in wild-type (*ACVRL1^wt/wt^*) iPSCs, resulting in a heterozygous 17 bp frameshift deletion in the *ACVRL1* gene [NG_009549.1:g.13707_13723del; NM_000020.3:c.1137_1153del]. After the generation of embryoid bodies (EBs), endothelial differentiation was induced via adding 4 ng/mL BMP4, 2% B27, and 10 ng/mL VEGF. Endothelial differentiation was monitored via immunocytochemistry. An analysis of 151 TGF-beta/BMP-related genes was performed via RT-qPCR through the use of mRNA derived from single iPS cell cultures as well as endothelial cells derived from EBs after endothelial differentiation. Differential TGF-beta/BMP gene expression was observed between *ACVRL1^wt/wt^* and *ACVRL1^wt/mut^* iPSCs as well as endothelial cells. EBs derived from CRISPR/Cas9-designed *ACVRL1* mutant HHT type 2 iPSCs, together with their isogenic wild-type iPSC counterparts, can serve as valuable resources for HHT type 2 in vitro studies.

## 1. Introduction

Vascular anomalies are a highly heterogeneous group of vascular tumors and malformations [1]. Among the vascular malformations, hereditary hemorrhagic telangiectasia (HHT, also known as Osler–Weber–Rendu disease) is linked to subgroups of capillary and arteriovenous malformations. HHT represents an autosomal dominant inherited disease with an incidence of about 1:5000. Genetically, HHT is characterized by the presence of mutations in genes involved in the TGF (transforming growth factor)-beta and BMP (bone morphogenic protein) signaling pathways [2,3]. In the vast majority of HHT cases, one of two genes, *ENG* (HHT1) or *ACVRL1* (HHT2), is mutated in a single allele, resulting in the haploinsufficiency of the respective heterozygous gene [4,5], leading to a disturbance of normal angiogenesis [6].

Clinically, HHT patients suffer from chronic and frequent nosebleeds, severely impacting their quality of life [7]; however, serious and life-threatening bleeding can also occur in other regions of the body, such as the gastrointestinal tract [8,9,10] and brain [11]. Since normal capillary bed formation between the arterious and venous arms of blood circulation is typically disturbed, leading to arteriovenous fistulas, HHT patients may also develop congestive heart failure due to an increased volume load on the heart [12]. There is no cure for HHT, so the development of new therapies is of high priority. Several HHT model systems have been developed for in vitro and in vivo investigations, such as HHT knockout mice [13,14]. In addition, HHT-patient-derived induced pluripotent stem cells (iPSCs) were generated and deployed for the in vitro analysis of angiogenesis [15,16], as well as to demonstrate successful in vitro CRISPR/Cas9-mediated repair of the respective HHT mutation [17]. Using CRISPR/Cas9, we induce an HHT-type-2-associated heterozygous mutation in normal iPSCs and analyze the consequences of this mutation on the TGF-beta/BMP signaling pathway in iPSC cultures as well as endothelial cells isolated from embryoid bodies.

## 2. Materials and Methods

### 2.1. iPSCs

Wild-type (wt) (*ACVRL1^wt/wt^*) iPSCs have been reported previously [18] and were kindly provided by Dr. Boris Greber (Catalent Cell and Gene Therapy, Langenfeld, Germany). iPSCs were grown on Matrigel (cat# 354230; Corning^®^ Matrigel^®^ Growth Factor Reduced (GFR) Basement Membrane Matrix, LDEV-free, Corning GmbH, Wiesbaden, Germany)-coated cell culture plates in Essential 8^TM^ Flex medium (cat# A2858501; Thermo Fisher Scientific, Darmstadt, Germany) supplemented with penicillin (final concentration of 5 U/mL) and streptomycin (final concentration of 5 μg/mL).

### 2.2. Design and Selection of sgRNAs

sgRNAs were designed using the ChopChop online tool (https://chopchop.cbu.uib.no), and four sgRNAs were selected for testing. The synthesis of sgRNAs was performed with the Guide-it sgRNA in vitro transcription kit and Guide-it IVT RNA clean-up kit (Takara Bio Inc., Shiga, Japan) according to the manufacturer’s protocol. Cas9-dependent in vitro cutting efficiency was evaluated with the Guide-it sgRNA screening kit (Takara Bio Inc.).

### 2.3. CRISPR/Cas9 ACVRL1 Knockout

Suspended in 390 μL of a hypoosmolar buffer (cat# 732-6007, Eppendorf, Hamburg, Germany), 2.4 × 10^5^ cells were co-transfected with 1.8 μg of sgRNA and 9 μg of Guide-it recombinant Cas9 protein (Takara Bio Inc.) via electroporation using the Multiporator System^®^ (Eppendorf) with a total volume of 400 μL in 2 mm cuvettes (cat# 4307000593, Eppendorf). The transfection (one pulse) parameters were pulse voltage (400 V) and pulse length (80 μs). After transfection, cell concentration was adjusted to 80 cells/10 mL using an Essential 8^TM^ Flex medium without antibiotics, and 100 µL of the cell suspension was added to each well of a 96-well plate. At least five 96-well plates were used for each sgRNA. Cell growth was monitored on a daily basis, and the appearance of single-cell clones (app. after 2 weeks) was documented. Well grown cell clones were transferred into 24-well plates and subsequently into 6-well plates. Testing for positive clones was performed with the Guide-it Mutation Detection Kit (Takara Bio Inc.) via the use of *ACVRL1*-specific primers (*ACVRL1*_forward: GTCCCACTGTTTCTCTCAGTCC; *ACVRL1*_reverse: CAAGCCTATGCTCTTAGCCACT) that enclose the assumptive mutation site. Positive clones that were susceptible to resolvase underwent Sanger sequencing (4baseLab, Reutlingen, Germany). The TA cloning of PCR-derived amplicons was performed for single-allele sequencing. The genotyping of iPSCs was performed at the DSMZ (Leibniz Institute—German Collection of Microorganisms and Cell Cultures GmbH, Braunschweig, Germany).

### 2.4. Western Blot Analysis

Whole-cell protein lysates were created through exposing cells to a lysis buffer (20 mmol/L Tris/HCl pH 7,5; 137 mmol/L NaCl; 10% Glycerol; 1% NP40; 2 mmol/L EDTA) supplemented with 100 μL/mL Proteaseinhibitor Cocktail for Mammalian Cell Extracts (cat# P8340; Sigma-Aldrich Inc., Saint Louis, MO, USA) and 50 μL/mL Phosphatase Inhibitor Cocktail 2 (cat# P5726; Sigma-Aldrich Inc.). Lysis was carried out for 60 min at 4 °C, and the supernatant was harvested after spinning the lysate (>12,000 g) for 10 min at 4 °C. Protein concentration was measured with the Bradford method (Bio-Rad Protein Assay Dye Reagent Concentrate; cat# 5000006; Bio-Rad Laboratories GmbH, Feldkirchen, Germany), and 20 µg of whole-cell protein lysate was separated in a 10% SDS polyacrylamide gel followed by being transferred onto a nitrocellulose membrane. Membranes were blocked for 20 min at RT in 3% skim milk/PBS, and an ACVRL1-specific rabbit polyclonal antibody (cat# PA5-14922; Invitrogen by Thermo Fisher Scientific), directed against the C-terminal region of human ACVRL1 (aa 474-503), was added to a final concentration of 2 µg/mL followed by incubation overnight at 4 °C. After washing membranes in a blocking buffer (2 × 5 min at RT), an HRP-coupled secondary antibody (0.8 µg/mL; cat# AP187P; goat anti-rabbit IgG antibody, HRP conjugate, species adsorbed; Merck KGaA, Darmstadt, Germany) was added and incubation was continued for 1 h at RT. After repeated washing, ACVRL1-specific bands were visualized with the enhanced chemiluminescence (ECL) method via exposing X-ray films. An antibody directed against GAPDH (0.2 µg/mL; sc-47724; Santa Cruz Biotechnology, Inc., Santa Cruz, CA, USA), followed by a secondary HRP-coupled antibody (0.8 µg/mL; cat# AP181P; goat anti-mouse IgG antibody, HRP conjugate, species adsorbed; Merck KGaA), served as a loading control. X-ray films were scanned and bands were quantified via the use of ImageJ software [19]. Similarly, antibodies directed against ENG (1:500, Dako, M3527, mouse), TGF beta RII (1:500, Tyr 424) (1:500, sc-17007, rabbit), TGF beta 1 (1:500, sc-146, rabbit), SMAD2/3 (1:500, sc-133098, mouse), and SMAD4 (1:500, sc-7966, mouse) were used in the WB analysis of iPSCs. The quantification of SMAD4 and TGF beta 1 signals was performed in the same manner as that described for ACVRL1.

### 2.5. Generation of Embryoid Bodies (EBs)

iPSCs were grown until they reached 80% confluence; 1.5 × 10^6^ iPSCs/10 mL E8-Flex media were added into 50 mL CEROtubes (OLS—OMNI Life Science GmbH & Co KG, Bremen, Germany). To generate embryoid bodies (EBs), iPSCs were incubated in a CERO 3D Incubator & Bioreactor system (OLS) in two steps: inoculation (settings: 60 rpm, rotate 2 s, pause 1 s, and duration of 12 h) and cultivation (settings: 80 rpm, rotate 1 s, pause 1 s, and duration of 144 h). EB formation was documented microscopically via the use of 25 µL of the EB suspension.

### 2.6. Endothelial Differentiation in EBs

To induce differentiation, EBs were continuously cultivated in a CERO 3D Incubator & Bioreactor system (settings: 80 rpm, rotate 1 s, and pause 2 s), but were changed into RPMI 1640 media (cat# 22400089, Gibco^TM^—Thermo Fisher Scientific) supplemented with Activin A (5 ng/mL; cat# 78001; STEMCELL Technologies Germany GmbH, Cologne, Germany), BMP4 (4 ng/mL; cat# AFL314E-010, R&D Systems, Minneapolis, MN, USA), 1% B27™ Supplement minus Insulin (cat# A1895601; Thermo Fisher Scientific), and 2 µmol/L CHIR-99021 (Laduviglusib) (cat# S2924; Selleck Chemicals LLC, Houston, TX, USA). Exactly 24 h later, the medium was exchanged for RPMI 1640 supplemented with 10 ng/mL VEGFA (vascular endothelial growth factor A) (cat# 78073.1; STEMCELL Technologies Germany GmbH), BMP4 (4 ng/mL), and B27^TM^ minus insulin (2%). Cultivation was continued for 15 days.

### 2.7. Isolation of Endothelial Cells from EBs

After the induction of endothelial differentiation as described above, EBs were harvested and exposed to a mixture of dispase (cat# 17105-041; Dispase II; Gibco^TM^—Thermo Fisher Scientific) and collagenase (cat# 17101-015; Collagenase Type II; Gibco^TM^—Thermo Fisher Scientific). After microscopic control of single-cell dissociation, cells were filtered through a 70 µm cell filter (Cell Strainer, BD Biosciences, Durham, NC, USA) in order to remove debris and subsequently washed in regular media (200 g, 4 min). The cell pellet was resuspended in regular media and incubated with superparamagnetic beads carrying an anti-CD31 antibody (Dynabeads^TM^ CD31 Endothelial Cell; cat# 11155D; Invitrogen by Thermo Fisher Scientific). CD31-positive (+) cells were bound in a magnetic field, and the negatively selected CD31-negative (−) cells were saved for further analyses. The magnetically bound CD31+ cells were washed twice, and the resulting cell/bead pellet, together with the CD31- pellet, was subjected to RNA extraction as well as quality control, as described below. The RNA integrity number (RIN) values were between 5.3 and 7.9. To validate the efficient enrichment of endothelial cells, quantitative RT-PCR was performed with *CD31*-specific primers (*CD31*_forward: CTGGACGGTGCAAAATGGGA; *CD31*_reverse: GTGCTGAGGCTTGACGTGAG), with *RPLP0* (*RPLP0*_forward: CCTTCTCCTTTGGGCTGGTCA; *RPLP0*_reverse: TCTGCAGACAGACACT) used as a housekeeping gene. The RNA from one experiment, which demonstrated the clear separation and enrichment of CD31+ cells, was used for a gene expression analysis, as described below.

### 2.8. Immunocytochemistry

One ml of the EB suspension was removed, and EBs were fixed in 4% paraformaldehyde (PFA) at 4 °C for 4 h and subsequently stored in PBS buffer. Anti-human CD31 directed mouse monoclonal antibodies (Dako—Agilent, Santa Clara, CA, USA) were used at a 1:100 dilution, followed by staining with an Alexa Fluor 488 antibody directed against mouse IgG (cat# A21202; Alexa Fluor^TM^ 488 donkey anti-mouse IgG (H+L); Thermo Fisher Scientific). Alexa-Fluor-647-coupled phalloidin (cat# 00041; Phalloidin, CF^®^647, Biotium—BIOZOL Diagnostica Vertrieb GmbH, Eching, Germany) was deployed to stain the F (filamentous)-actin cytoskeleton of the cells. Anti-smooth muscle actin (SMA) was detected with an Alexa-555-coupled anti-SMA rabbit monoclonal antibody (cat# ab202509; Alexa Fluor^®^ 555 Anti-alpha smooth muscle Actin antibody (clone EPR5368); Abcam plc, Cambridge, UK). DAPI was used to counterstain the nucleus. Microscopic analysis and documentation were carried out with a confocal laser scanning microscope (Leica SP8i, Microscopy Core Facility, Faculty of Medicine, Philipps-Universität Marburg, Marburg, Germany).

### 2.9. Gene Expression Analysis

*ACVRL1^wt/wt^* and *ACVRL^wt/mut^* iPSCs were grown until reaching 80% confluence, followed by detachment (ReLeSR^TM^, STEMCELL Technologies Germany GmbH) and RNA extraction via the use of the RNeasy Mini kit (Qiagen, Hilden, Germany). RNA concentration and quality were assessed with an Implen NanoPhotometer^®^ NP80 (Implen, Inc.; Westlake Village, CA, USA) and a 2100 Bioanalyzer system (Eukaryote total RNA Nano assay; Agilent, Santa Clara, CA, USA; Genomics Core Facility, Faculty of Medicine, Philipps-Universität Marburg, Marburg, Germany). The RIN values were between 9.4 and 9.9. For reverse transcription, the RT^2^ first strand kit, and for real-time PCR the RT^2^ SYBR Green qPCR Mastermix (both Qiagen) were used. Differences in the expression of the genes involved in the TGF-beta/BMP signaling pathways were assessed with RT^2^ Profiler™ PCR Arrays. RT^2^ Profiler™ PCR Arrays Human TGF-beta/BMP Signaling (PAHS-035YA) and Human TGF-beta Signaling Targets (PAHS-235ZA) (Qiagen Sciences, Germantown, MD, USA) were used according to the manufacturer’s instructions. The analysis was performed via the use of the software provided by the manufacturer.

### 2.10. Statistical Analysis

Tests were performed with the Prism 6.0 software (GraphPad Software, Inc., San Diego, CA, USA). The data of the Western blot analysis were analyzed with a paired two-tailed Student’s *t*-test. Statistics for RT^2^ Profiler analyses were carried out using a two-tailed, unpaired Student’s t-test. A one-tailed, unpaired Student’s *t*-test was used to compare CD31 mRNA expression levels after magnetic cell separation (MACS). Data represent the mean ± SD, with *p* < 0.05 considered statistically significant.

## 3. Results

### 3.1. ACVRL1 Knockout Using CRISPR/Cas9

**Figure 1:**
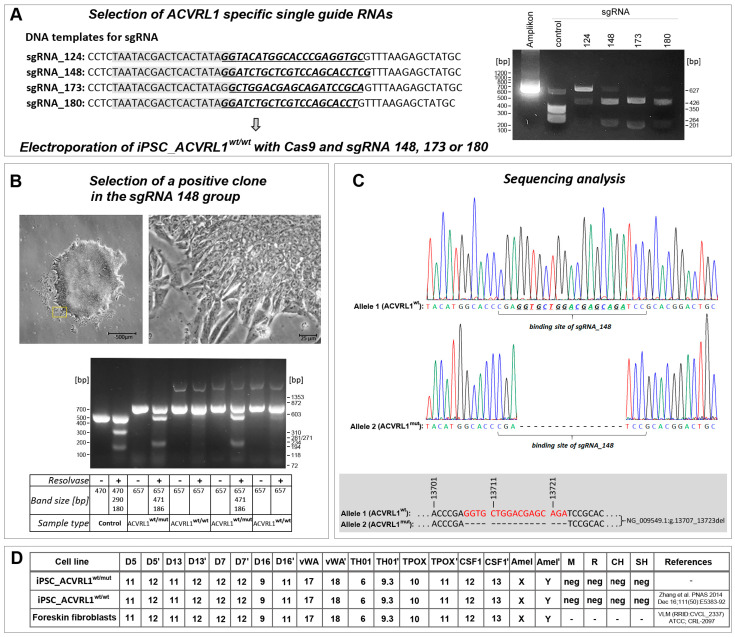
Generation and validation of iPS *ACVRL1^wt/mut^* cells. (**A**) Three of four candidate sgRNAs demonstrated efficient cleavage in vitro and were selected for transfection into iPS *ACVRL1^wt/wt^* cells. The gene-specific sequence is underlined and depicted in bold as well as italics. The T7 promoter region used for in vitro transcription is highlighted in grey. (**B**) A positive clone was isolated from iPSCs transfected with sgRNA148. (**C**) Sequence analysis revealed a frameshift deletion of 17 base pairs in one allele (exon 8) of the *ACVRL1* gene. (**D**) The identity of the new mutant *ACVRL1^wt/mut^* iPS cell line was validated via genotyping and comparison with the cells of origin (M—mouse; R—rat; CH—Chinese hamster; and SH—Syrian hamster) [18].

**Figure 2:**
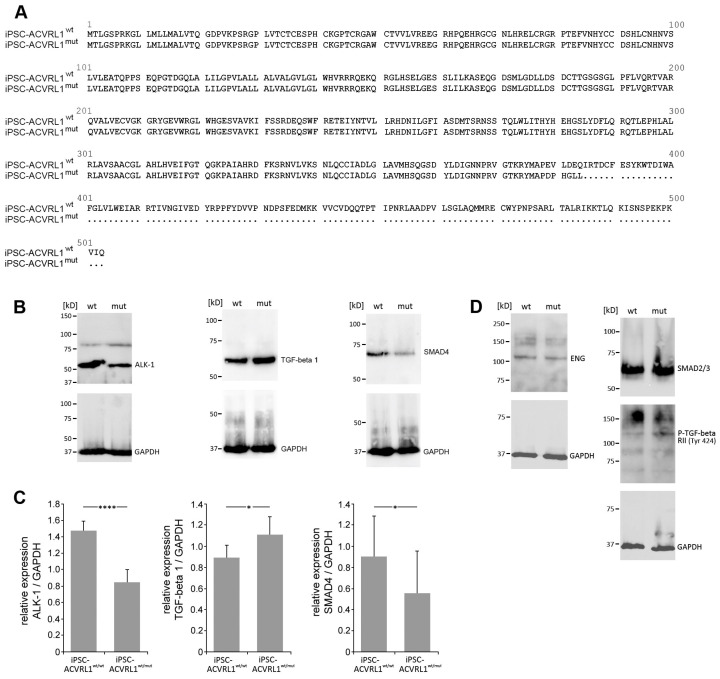
Effect of the heterozygous *ACVRL1* mutation on the expression levels of associated signaling molecules. (**A**) The mutant allele encodes for a C-terminally truncated ALK-1 protein. (**B**,**C**) Differing expression levels of ALK-1, TGF-beta 1 (corresponding to the precursor form), and SMAD4 between wt and mutant iPSCs. (**D**) No obvious differences in protein expression levels for ENG, SMAD2/3, and p-TGF-beta RII (Tyr 424) between wt and mutant iPSCs. (* *p* < 0.05; **** *p* < 0.0001).

Four single-guide (sg) RNAs were tested in vitro in the presence of a recombinant Cas9 protein for their ability to cut a selected DNA test sequence of the *ACVRL1* gene. Three sgRNAs (sgRNA_148, sgRNA_173, and sgRNA_180) exhibited high cleavage efficiency (Figure 1A) and were subsequently selected for deployment with iPS wt cells. iPSCs were transfected via electroporation with Cas9 together with one of the three sgRNAs. A positive clone could be derived from iPSCs receiving sgRNA148 (Figure 1B). A sequence analysis revealed a heterozygous frameshift deletion of 17 base pairs (NG_009549.1:g.13707_13723del, corresponding to NM_000020.3:c.1137_1153del) (Figure 1C). Both the parental *ACVRL^wt/wt^* and *ACVRL^wt/mut^* iPS cell lines were genotyped, confirming their identity and their origin from fibroblast cells, which were used for reprogramming into iPSCs, as reported earlier [18] (Figure 1D). After genotyping and a comparison with the STR database, we noticed the existence of another wt iPS cell line with the same genotype. Here, the authors used the same fibroblast resource for reprogramming [20] as that reported by Zhang et al. [18]. To evaluate ACVRL1 protein expression levels, an antibody directed against the C-terminus of the wt ACVRL1 protein was deployed, not capable of recognizing the predicted protein product of the mutant *ACVRL1* allele due to the loss of the relevant C-terminal portion in the protein (Figure 2A). A significant reduction in ACVRL1 wt protein expression could be observed in heterozygous mutant iPSCs compared to wt iPSCs. Similarly, SMAD4 also appeared to be downregulated in mutant iPSCs, whereas TGF-beta 1 (corresponding to the precursor form) appeared to be upregulated (Figure 2B). These three proteins were further quantified, demonstrating significant dysregulation (Figure 2C). The expression levels of ENG, SMAD2/3, and p-TGF-beta RII (Tyr 424) did not exhibit notable differences between both iPS cell lines (Figure 2D). Furthermore, using antibodies directed against BMP9, SMADs 1, 5, and 8, in our hands, did not produce evaluable bands.

### 3.2. Effect of Heterozygous ACVRL1 Knockout on the Expression of TGF-Beta and BMP Signaling Molecules

**Figure 3:**
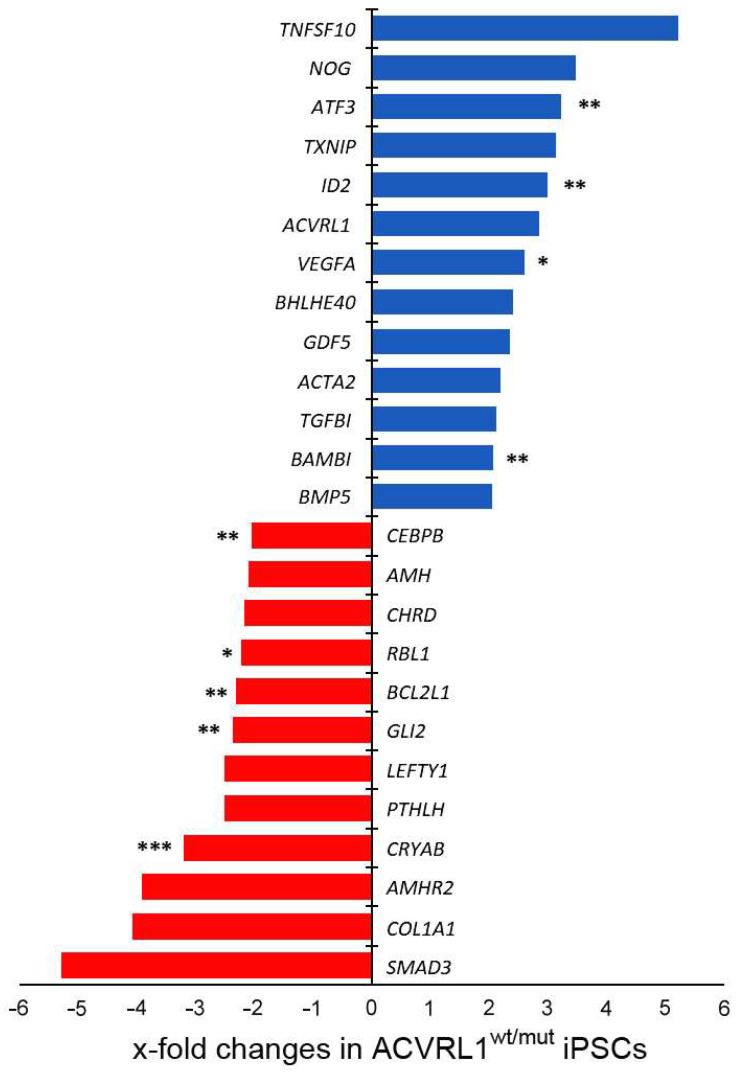
Differentially regulated genes in iPS *ACVRL1^wt/mut^* cells. Differences between iPS *ACVRL1^wt/wt^* and *ACVRL1^wt_mut^* cells (test sample: *n* = 3 for all groups) were analyzed via the use of an unpaired Student’s *t*-test. Statistical differences: * *p* < 0.05; ** *p* < 0.01; and *** *p* < 0.001. *Abbreviations—ACVRL1: Activin A receptor type II-like 1 (NM_000020); AMH: Anti-Mullerian hormone (NM_000479); AMHR2: Anti-Mullerian hormone receptor, type II (NM_020547); ATF3: Activating transcription factor 3 (NM_001674); BAMBI: BMP and activin membrane-bound inhibitor homolog (Xenopus laevis) (NM_012342); BCL2L1: BCL2-like 1 (NM_138578); BHLHE40: Basic helix-loop-helix family, member e40 (NM_003670); BMP5: Bone morphogenetic protein 5 (NM_021073); CREBBP: CREB binding protein (NM_004380); CHRD: Chordin (NM_003741); COL1A1: Collagen, type I, alpha 1 (NM_000088); CRYAB: Crystallin, alpha B (NM_001885); GDF5: Growth differentiation factor 5 (NM_000557); GLI2: GLI family zinc finger 2 (NM_005270); ID2: Inhibitor of DNA binding 2, dominant negative helix-loop-helix protein (NM_002166); LEFTY1: Left-right determination factor 1 (NM_020997); NOG: Noggin (NM_005450); PTHLH: Parathyroid hormone-like hormone (NM_002820); RBL1: Retinoblastoma-like 1 (p107) (NM_002895); SMAD3: SMAD family member 3 (NM_005902); TGFBI: Transforming growth factor, beta-induced, 68kDa (NM_000358); TNFSF10: Tumor necrosis factor (ligand) superfamily, member 10 (NM_003810); TXNIP: Serine/threonine kinase 38 like (NM_006472); and VEGFA: Vascular endothelial growth factor A (NM_003376). For a complete gene list, please see Table S1 in the Supplementary Materials*.

To evaluate the consequences of heterozygous *ACVRL1* knockout on TGF-beta and BMP signaling molecules, we performed quantitative RT-PCR on a total of 151 TGF-beta- and BMP-signaling-related genes. Genes that were more than two-fold up- or downregulated in *ACVRL1^wt/mut^* iPSCs are shown in Figure 3. Interestingly, among other genes, *ACVRL1* transcripts appeared upregulated in *ACVRL1^wt/mut^* iPSCs, possibly representing a compensatory mechanism due to the haploinsufficiency of the *ACVRL1* gene. Additionally, the significant upregulation of VEGFA in mutant iPSCs is noteworthy, since VEGF was shown to be associated with HHT disease [21].

### 3.3. Generation of Embryoid Bodies (EBs) from ACVRL1^wt/wt^ and ACVRL1^wt/mut^ iPSCs

**Figure 4:**
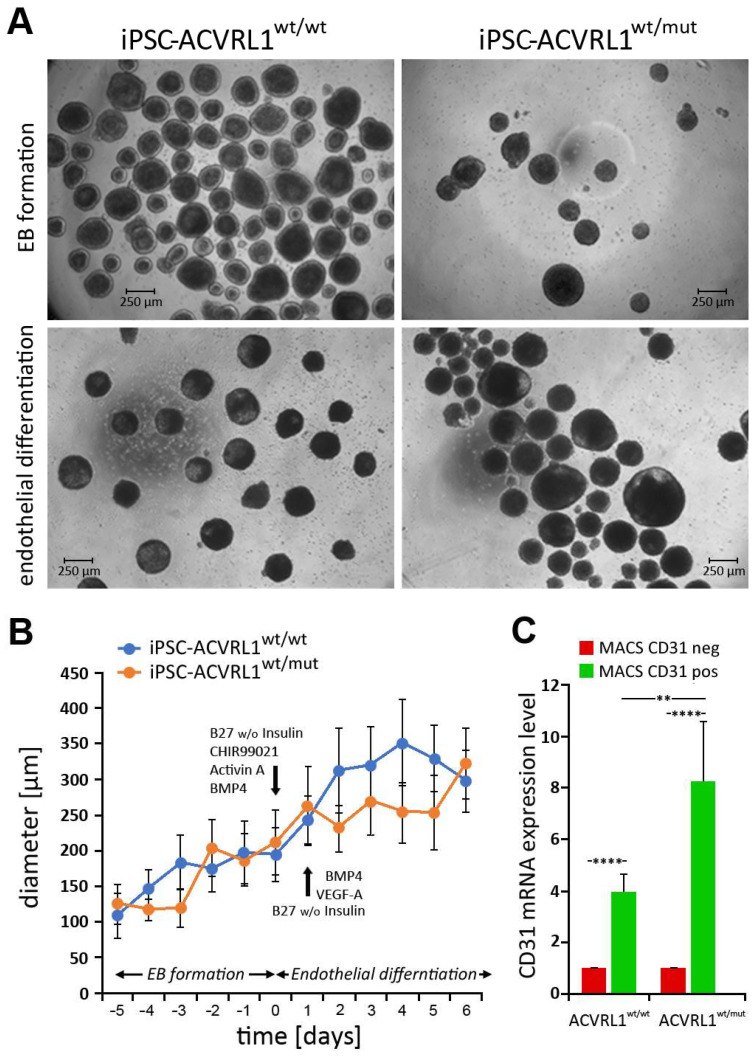
Generation of embryoid bodies (EBs). (**A**) EB formation from *ACVRL1^wt/wt^* and *ACVRL1^wt/mut^* iPSCs growing under nonadherent conditions via the use of a CERO 3D Incubator & Bioreactor. (**B**) The course of EB growth was assessed via monitoring the EB diameter. (**C**) After endothelial differentiation, CD31+ endothelial cells were detected by magnetic activated cell sorting (MACS) and subsequent RT-qPCR in both types of EBs. (Measurements: *n* = 4; evaluation method: one-tailed, unpaired Student´s *t*-test; and statistical differences: ** *p* < 0.01 and **** *p* < 0.0001.)

*ACVRL1^wt/wt^* and *ACVRL1^wt/mut^* iPSCs were cultured under nonadherent conditions to generate embryoid bodies (EBs) (Figure 4A). After EBs reached a diameter of around 200 µm, endothelial differentiation was induced. The growth of EBs was monitored, and the diameter of EBs was documented. Here, no significant differences between the diameters of *ACVRL1^wt/wt^*- and *ACVRL1^wt/mut^*-iPSC-derived EBs were noted (Figure 4B). Since endothelial cells represent the dominant structure of the pathomorphological correlate in HHT, we were interested in comparing endothelial cells derived from *ACVRL1* wt and mutant EBs. The dissociation of differentiated EBs into single cells and the isolation of CD31+ as well as CD31- cells via MACS were performed as described in the Materials and Methods section. MACS-isolated CD31+ cells as well as negative selected CD31- cells were analyzed via RT-qPCR with regard to their CD31 expression levels, thereby confirming successful cell separation. A highly significant enrichment of CD31+ cells could be achieved after MACS (Figure 4C). A single experiment for the purpose of feasibility, comparing 151 TGF-beta- and BMP-signaling-related genes in wt and mutant ECs, is shown in Supplementary Figure S1.

### 3.4. Induction of Endothelial Differentiation in Embryoid Bodies (EBs) Derived from ACVRL1^wt/wt^ and ACVRL1^wt/mut^ iPSCs

**Figure 5:**
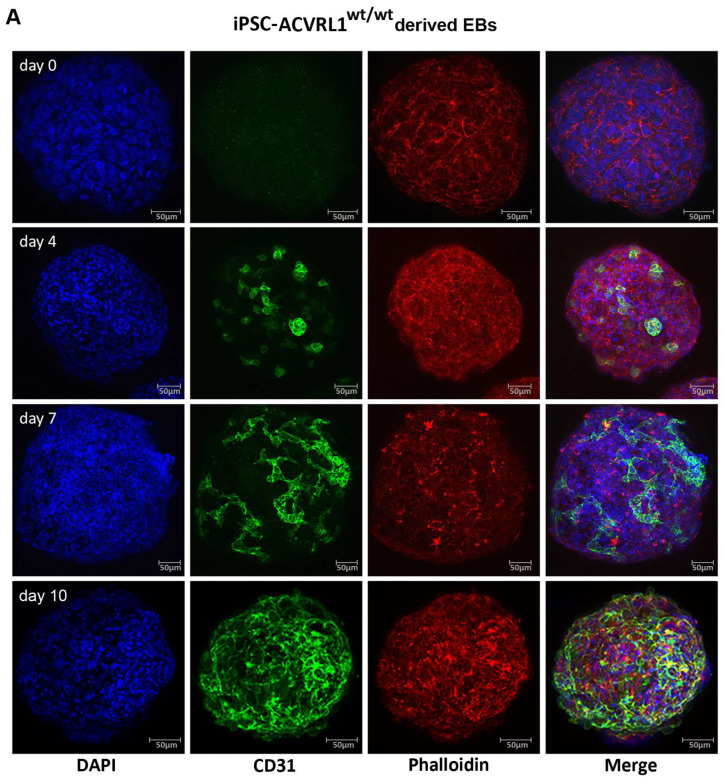

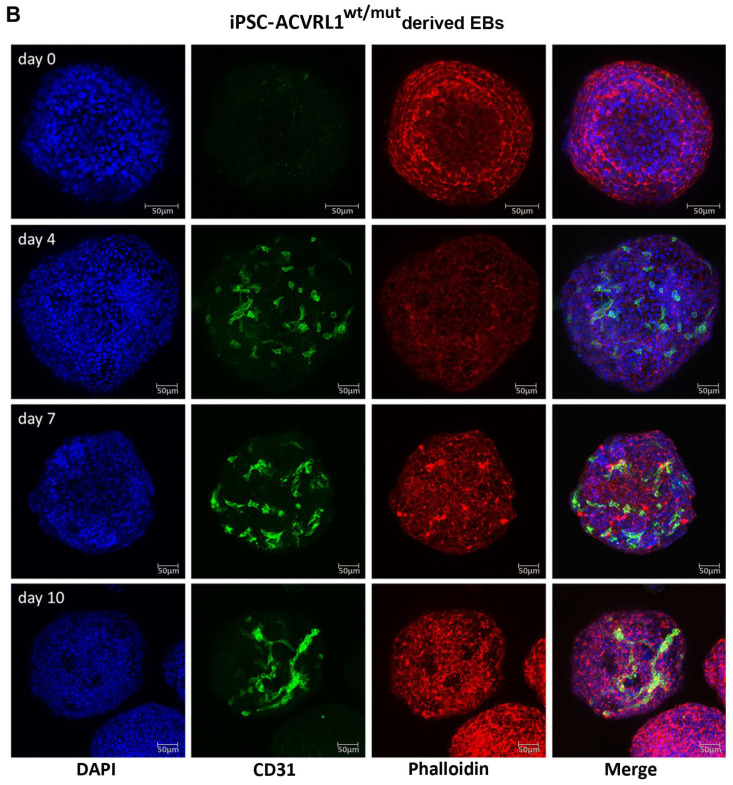

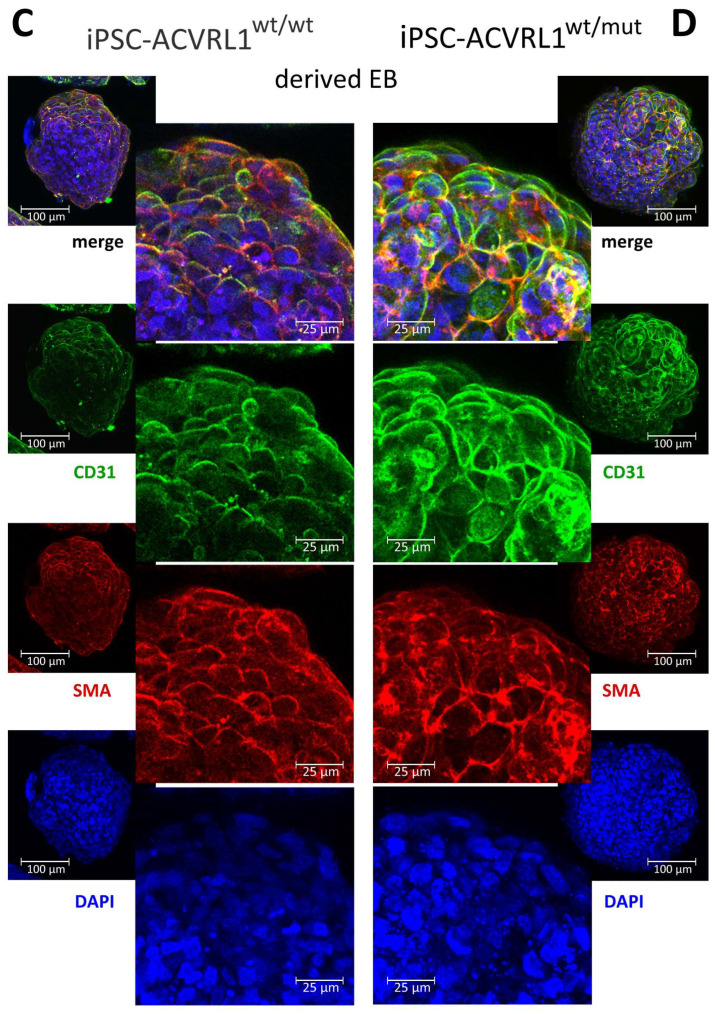
Induction of endothelial differentiation in EBs. Depicted are confocal microscopy images of (**A**) *ACVRL1^wt/wt^* and (**B**) *ACVRL1^wt/mut^* EBs documenting the appearance of CD31+ cells after endothelial induction. Phalloidin was used to highlight the F (filamentous)-actin cytoskeleton, whilst DAPI was used to stain the nucleus. (**C**,**D**) Staining of EBs with an antibody directed against smooth muscle actin to detect possible pericyte structures.

The endothelial differentiation of EBs was monitored via the confocal laser scanning microscopy of fixed EBs stained with the endothelial marker CD31. After the induction of endothelial differentiation at day 0, CD31+ cells appear in the EBs, which start to interconnect with each other (Figure 5A,B). At this time, we are not able to quantify the EB images; however, when comparing iPSC-*ACVRL1^wt/wt^*- and iPSC-*ACVRL1^wt/mut^*-derived EBs, the CD31+ structures in mutant EBs resemble dilated vessels in a reduced capillary bed—as is typical for HHT patients. Staining with an antibody directed against SMA aimed to detect possible pericyte structures (Figure 5C,D).

## 4. Discussion

HHT is a rare disorder. Therefore, biological materials derived from HHT patients for research purposes are highly limited. This is even more so the case as surgical procedures, which generate HHT biomaterials such as tissue specimens, are only performed occasionally and avoided, as the removal of tissues is a trauma that might result in the development of new telangiectasias [22]. Against this background, HHT model systems are of the utmost relevance to research groups working on HHT. Important HHT animal models, such as transgenic mice [13,14,23] and a zebrafish model [24], exist, but there is an increasing need for human model systems as they are expected to better reflect the situation in HHT patients, e.g., since both clinical manifestations of mutations and drug effects on animals are often different to those in humans. With the advent of powerful genome editing techniques, particularly CRISPR/Cas9, which allow for the knockout or even repair of genes, focus has been devoted to also deploying these techniques in HHT research.

In 2010, a group at the Leiden University Medical Center, led by Dr. Mummery, reported the generation of an iPS cell line derived from the skin fibroblasts of an HHT patient [16]. Subsequently, the portfolio of available HHT iPSCs was expanded by various research groups in the field [17,25]. In 2020, Bouma and colleagues reported about the generation of two iPS cell lines derived from fibroblasts of a HHT2 patient carrying a heterozygous 18 bp in frame deletion in exon 8 of the *ACVRL1* gene [17]. The authors used CRISPR/Cas9 to repair this mutation, thereby generating two isogenic iPS cell line pairs. Having such cell lines, which, except for the region of the mutation, are genetically identical, carries the promise of enabling more precise HHT research. Recent studies deploying such isogenic iPS cell lines, which were derived from a patient with a rare mosaic HHT1 mutation in the *ENG* gene, described vascular defects associated with the respective HHT1 mutation [15]. Interestingly, the CRISPR/Cas9-induced 17 bp frameshift deletion mutation in exon 8 of the *ACVRL1* gene in the iPSC line presented in our study is located directly next to the 18 bp in-frame deletion mutation found in patient-derived iPSCs as reported by Bouma and coworkers [17]. It will be interesting to compare both mutant iPSCs regarding the consequences of their mutations for HHT development.

Furthermore, there are several reports concerning frameshift mutations in exon 8 of the *ACVRL1* gene as found in the *ACVRL1* mutation database (https://arup.utah.edu), all of them being classified as pathogenic (e.g., c.1061_1068del, c.1073del, c.1102_1105del, c.1107_1108del, c.1118del, and c.1215del) [5,26]. These reports further support the clinical significance of the CRISPR/Cas9-designed mutant (c.1137_1153del) iPS cell line for HHT research, as reported in our study.

Several candidate genes were found to be associated with HHT [21]. Here, VEGF seems to play a major role in HHT disease progression. Initially, it was observed that VEGF serum and plasma levels were elevated in HHT patients [21,27]. Subsequent studies evaluated the consequences of VEGF inhibition in HHT patients. VEGF inhibitors, such as the anti-VEGF antibody bevacizumab, were shown to be effective in treating hepatic vascular malformations in addition to nose and gastrointestinal bleeding in HHT patients [28,29,30,31,32,33,34]. In our study, VEGFA was found to be elevated in *ACVRL1^wt/mut^* iPSCs. Using EBs and ECs derived from *ACVRL1* wt and mutant iPSCs could help in investigating the effects of VEGF inhibitors and other drugs on vasculogenesis, as well as in the comparison of differences between wt and mutant vessel formation.

Candidate genes identified in the present study, need to be further characterized and validated, followed by in vivo evaluations regarding their possible roles in HHT2 disease.

In this context, it is important to mention that a reduction in ACVRL1 (ALK1) results in enhanced pathological vasculogenesis [35,36]. Although, in our study, *ACVRL1* transcripts appeared to be upregulated in *ACVRL1^wt/mut^* iPSCs, likely as a response to the loss of function of one allele of the *ACVRL1* gene, wt protein expression in *ACVRL1^wt/mut^* iPSCs dropped to nearly half of the values observed for *ACVRL1^wt/wt^* iPSCs, confirming the haploinsufficiency of ACVRL1 at the protein level.

In recent years, attention has been given to the so-called second-hit hypothesis in HHT. The assumption here is that, in order to achieve a full clinical HHT phenotype, a second genetic hit (somatic mutation) at the wt allele of the *ACVRL1^wt/mut^* gene is required and that the classical single HHT germline mutation alone possibly is not sufficient to produce a full blown HHT phenotype [37,38,39,40,41]. This was intriguingly and convincingly demonstrated by Snellings and coworkers: next to the existence of an inherited germline mutation within one allele of an HHT-related gene, they demonstrated the additional presence of a somatic mutation in the other allele of the same gene when performing a next-generation sequencing analysis of telangiectasias. They proposed that a two-hit event (germline mutation and somatic mutation) is required to produce a typical HHT phenotype, such as telangiectasias, thereby questioning the notion of haploinsufficiency [41]. In addition, a recent report suggests that even a third hit, such as trauma, could contribute to a clinical HHT phenotype [22]. The syngeneic pair of wt and mutant iPSCs presented in our study allows for studying the effect of only a single *ACVRL1* mutation on vasculogenesis, since a concomitant second-hit mutation is largely excluded when performing CISPR/Cas9 *ACVRL1* gene knockout on wt iPSCs. Furthermore, future studies will use the mutant iPS cell line described in our study to induce an additional pathogenic mutation in the wt *ACVRL1* allele, thereby generating a cell line with a biallelic loss of the respective gene. Comparing such HHT2-related engineered iPS cell lines with other existing patient-derived HHT2 iPS cell lines could explain differences in the vasculo- and angiogenesis between both types of iPS cell lines, which could help to better understand how specific mutations affect HHT2 disease progression. The two iPS cell lines reported in our study could therefore significantly add to the portfolio of iPS cells for HHT research.

## 5. Conclusions

Here, we report the generation and validation of a heterozygous *ACVRL1* knockout iPS cell line and its potential use in HHT2 research. In vitro studies trying to mimic the HHT phenotype are complex since the view prevails that, next to a specific HHT mutation, a second genetic hit is required to allow for the development of a true HHT phenotype. By having a pair of iPS cell lines, a wt one and its syngeneic *ACVRL1* mutant counterpart in which only the specific HHT gene is mutated in one allele, the presence of a second hit is largely excluded. This pair of iPSCs, in conjunction with other existing HHT iPSCs, could therefore help to elucidate which phenotype is dependent on a specific HHT mutation only and which phenotype requires additional mutations (second hits).

## Data Availability

Not applicable.

## Supplementary Materials

The following supporting information can be downloaded at: https://www.mdpi.com/article/10.3390/cells12121600/s1, Supplementary Figure S1: Differentially regulated genes in CD31+ cells isolated from *ACVRL1^wt/wt^* and *ACVRL1^wt/mut^* EBs. Table S1: Results of RT^2^ Profiler PCR Array: Human TGFβ Signaling Pathway Plus (Qiagen^®^ PAHS-035YA). Table S2: Results of RT^2^ Profiler PCR Array: Human TGFβ Signaling Targets (Qiagen^®^ PAHS-235ZA). Uncropped Western blot images.
