# Supplementary material for: Generation of a Syngeneic Heterozygous ACVRL1(wt/mut) Knockout iPS Cell Line for the In Vitro Study of HHT2-Associated Angiogenesis
Source: Cells. 2023 Jun 10;12(12):1600. doi: 10.3390/cells12121600 (PMC10297623; doi:10.3390/cells12121600)

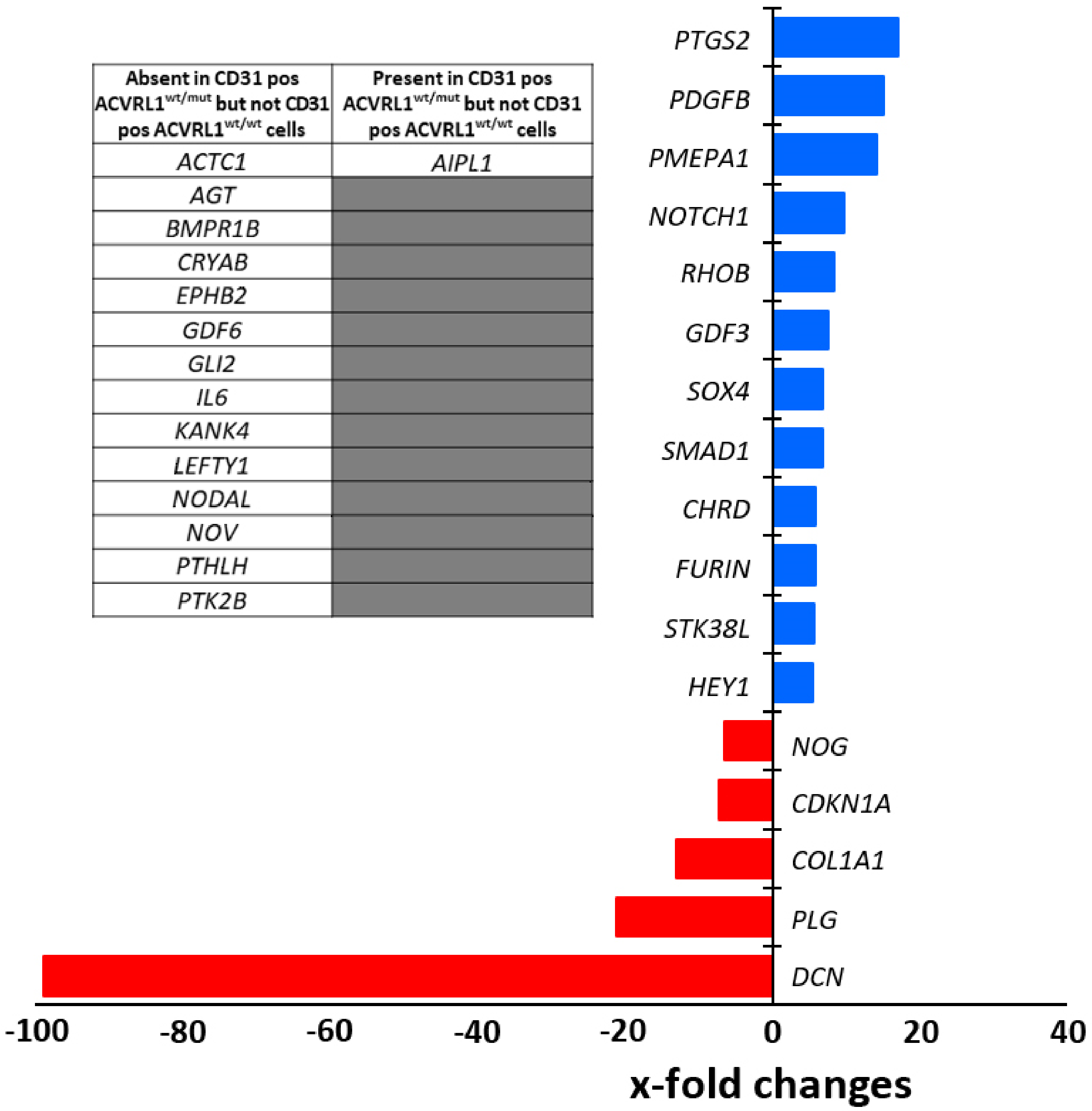

Absent in CD31 pos ACVRL1$^{wt/mut}$ but not CD31 pos ACVRL1$^{wt/wt}$ cells	Present in CD31 pos ACVRL1$^{wt/mut}$ but not CD31 pos ACVRL1$^{wt/wt}$ cells
ACTC1	*AIPL1*
AGT	
BMPR1B	
CRYAB	
EPHB2	
GDF6	
GLI2	
IL6	
KANK4	
LEFTY1	
NODAL	
NOV	
PTHLH	
PTK2B	

Supplementary Figure S1. Differentially regulated genes in CD31+ cells isolated from *ACVRL1*$^{wt/wt}$ and *ACVRL1*$^{wt/mut}$ EBs. Differences (> 5 fold) in mRNA expression between CD31+ cells derived from *ACVRL1*$^{wt/wt}$ and *ACVRL1*$^{wt/mut}$ EBs are depicted in the graph. The table summarizes mRNA expression of genes of the TGF-beta Signaling Pathway and associated targets present in CD31+ *ACVRL1*$^{wt/wt}$ but not in CD31+ *ACVRL1*$^{wt/mut}$ cells (absent) or exclusively present in CD31+ *ACVRL1*$^{wt/mut}$ cells (present). *Abbreviations (includes only the regulated genes listed in the graph. For all other genes, see the gene list in the supplement): CDKN1A: Cyclin-dependent kinase inhibitor 1A (p21, Cip1) (NM_000389); CHRD: Chordin (NM_003741); COL1A1: Collagen, type I, alpha 1 (NM_000088); DCN: Decorin (NM_001920); FURIN: Furin (paired basic amino acid cleaving enzyme) (NM_002569); GDF3: Growth differentiation factor 3 (NM_020634); HEY1: Hairy/enhancer-of-split related with YRPW motif 1 (NM_012258); NOG: Noggin (NM_005450); NOTCH1: Notch 1 (NM_017617); PDGFB: Platelet-derived growth factor beta polypeptide (NM_002608); PMEPA1: Prostate transmembrane protein, androgen induced 1 (NM_020182); PTGS2: Prostaglandin-endoperoxide synthase 2 (prostaglandin G/H synthase and cyclooxygenase) (NM_000963); RHOB: Ras homolog gene family, member B (NM_004040); SMAD1: SMAD family member 1 (NM_005900); STK38L: Serine/threonine kinase 38 like (NM_015000). The data is derived from a single experiment to demonstrate feasibility of gene analysis in MACS isolated CD31+ endothelial cells.*

Table S1. Results of RT^2 Profiler PCR Array: Human TGFβ Signaling Pathway Plus (Qiagen® PAHS-035YA)

Well	RefSeq	Symbol	AVG ΔCt		2^-ΔCt		fold change	*t*-test p	fold Up- or Down-Regulation
			ACVRL1-wt/mut	ACVRL1-wt/wt	ACVRL1-wt/mut	ACVRL1-wt/wt	ACVRL1-wt/mut to wt/wt	value	ACVRL1- wt/mut to wt/wt
A01	NM_001105	ACVR1	5,692	5,116	0,019	0,029	0,671	0,069	-1,491
A02	NM_001616	ACVR2A	5,249	4,933	0,026	0,033	0,803	0,082	-1,246
A03	NM_000020	ACVRL1	10,384	11,902	0,001	0,000	2,862	0,198	2,862
A04	NM_000479	AMH	10,010	8,956	0,001	0,002	0,482	0,399	-2,077
A05	NM_020547	AMHR2	12,378	10,419	0,000	0,001	0,257	0,301	-3,886
A06	NM_001675	ATF4	0,187	0,362	0,879	0,778	1,129	0,294	1,129
A07	NM_012342	BAMBI	1,717	2,771	0,304	0,147	2,076	0,003	2,076
A08	NM_199173	BGLAP	5,647	6,025	0,020	0,015	1,300	0,264	1,300
A09	NM_006129	BMP1	6,333	6,062	0,012	0,015	0,828	0,315	-1,207
A10	NM_001200	BMP2	6,404	6,666	0,012	0,010	1,199	0,614	1,199
A11	NM_001201	BMP3	10,623	11,114	0,001	0,000	1,405	0,263	1,405
A12	NM_130851	BMP4	5,886	6,303	0,017	0,013	1,335	0,062	1,335
B01	NM_021073	BMP5	10,022	11,049	0,001	0,000	2,038	0,350	2,038
B02	NM_001718	BMP6	7,569	8,001	0,005	0,004	1,350	0,493	1,350
B03	NM_001719	BMP7	3,915	3,946	0,066	0,065	1,022	0,828	1,022
B04	NM_133468	BMPER	6,643	7,088	0,010	0,007	1,361	0,373	1,361
B05	NM_004329	BMPR1A	2,876	2,712	0,136	0,153	0,893	0,037	-1,120
B06	NM_001203	BMPR1B	9,381	9,214	0,001	0,002	0,891	0,691	-1,122
B07	NM_000389	CDKN1A	2,305	3,234	0,202	0,106	1,905	0,561	1,905
B08	NM_004064	CDKN1B	4,848	4,789	0,035	0,036	0,960	0,713	-1,042
B09	NM_004936	CDKN2B	11,431	10,865	0,000	0,001	0,676	0,908	-1,480
B10	NM_003741	CHRD	8,765	7,668	0,002	0,005	0,468	0,289	-2,138
B11	NM_000088	COL1A1	5,013	2,992	0,031	0,126	0,246	0,068	-4,059
B12	NM_000089	COL1A2	3,008	2,975	0,124	0,127	0,978	0,783	-1,023
C01	NM_001920	DCN	7,839	7,121	0,004	0,007	0,608	0,309	-1,645
C02	NM_001423	EMP1	13,019	13,629	0,000	0,000	1,526	0,356	1,526
C03	NM_000118	ENG	7,516	7,490	0,005	0,006	0,982	0,782	-1,018
C04	NM_015675	GADD45B	3,504	4,094	0,088	0,059	1,506	0,047	1,506
C05	NM_016204	GDF2	12,802	12,679	0,000	0,000	0,919	0,710	-1,089
C06	NM_020634	GDF3	2,146	2,614	0,226	0,163	1,383	0,166	1,383
C07	NM_000557	GDF5	12,209	13,446	0,000	0,000	2,357	0,116	2,357
C08	NM_001001557	GDF6	9,940	10,398	0,001	0,001	1,373	0,351	1,373
C09	NM_182828	GDF7	10,232	11,176	0,001	0,000	1,924	0,056	1,924
C10	NM_173849	GSC	5,742	5,032	0,019	0,031	0,611	0,528	-1,636
C11	NM_014685	HERPUD1	5,685	5,698	0,019	0,019	1,009	0,917	1,009
C12	NM_002165	ID1	2,015	1,822	0,247	0,283	0,875	0,515	-1,143
D01	NM_002166	ID2	2,013	3,555	0,248	0,085	2,912	0,004	2,912
D02	NM_001550	IFRD1	5,065	4,312	0,030	0,050	0,593	0,208	-1,685
D03	NM_000618	IGF1	12,004	12,222	0,000	0,000	1,163	0,523	1,163
D04	NM_000598	IGFBP3	6,785	6,236	0,009	0,013	0,684	0,027	-1,462
D05	NM_000600	IL6	9,400	8,812	0,001	0,002	0,666	0,267	-1,503
D06	NM_002191	INHA	9,252	9,448	0,002	0,001	1,146	0,931	1,146
D07	NM_020997	LEFTY1	3,469	2,156	0,090	0,224	0,403	0,567	-2,484
D08	NM_000627	LTBP1	2,967	3,402	0,128	0,095	1,352	0,076	1,352
D09	NM_002467	MYC V-myc	1,962	2,803	0,257	0,143	1,792	0,135	1,792
D10	NM_018055	NODAL	3,223	2,273	0,107	0,207	0,518	0,312	-1,931
D11	NM_005450	NOG	6,388	8,188	0,012	0,003	3,483	0,057	3,483
D12	NM_002608	PDGFB	7,970	8,911	0,004	0,002	1,920	0,523	1,920
E01	NM_002658	PLAU	3,985	4,834	0,063	0,035	1,801	0,049	1,801
E02	NM_005900	SMAD1	4,807	4,436	0,036	0,046	0,773	0,275	-1,294
E03	NM_005901	SMAD2	3,143	2,936	0,113	0,131	0,867	0,040	-1,154

E04	NM_005902	SMAD3	8,704	7,364	0,002	0,006	0,395	0,209	-2,531
E05	NM_005359	SMAD4	3,879	3,491	0,068	0,089	0,764	0,014	-1,309
E06	NM_005903	SMAD5	3,561	3,309	0,085	0,101	0,840	0,031	-1,191
E07	NM_005904	SMAD7	3,638	3,221	0,080	0,107	0,749	0,153	-1,334
E08	NM_020429	SMURF1	5,097	4,512	0,029	0,044	0,666	0,002	-1,501
E09	NM_007315	STAT1	3,854	3,728	0,069	0,075	0,916	0,031	-1,091
E10	NM_000660	TGFB1	4,246	4,120	0,053	0,058	0,917	0,574	-1,091
E11	NM_015927	TGFB1I1	8,494	8,046	0,003	0,004	0,733	0,064	-1,365
E12	NM_003238	TGFB2	7,446	7,801	0,006	0,004	1,279	0,398	1,279
F01	NM_003239	TGFB3	9,476	9,189	0,001	0,002	0,819	0,643	-1,221
F02	NM_000358	TGFBI	6,216	7,298	0,013	0,006	2,118	0,064	2,118
F03	NM_004612	TGFBR1	4,340	4,147	0,049	0,056	0,875	0,407	-1,143
F04	NM_003242	TGFBR2	6,236	5,749	0,013	0,019	0,714	0,010	-1,401
F05	NM_003243	TGFBR3	7,165	7,446	0,007	0,006	1,214	0,152	1,214
F06	NM_003244	TGIF1	2,235	1,926	0,212	0,263	0,808	0,001	-1,238
F07	NM_003246	THBS1	4,490	3,738	0,045	0,075	0,594	0,090	-1,685
F08	NM_003810	TNFSF10	10,012	9,343	0,001	0,002	0,629	0,117	-1,591
F09	NM_005159	ACTC1	7,072	4,948	0,007	0,032	0,230	0,004	-4,356
F10	NM_001204	BMPR2	4,249	4,355	0,053	0,049	1,076	0,253	1,076
F11	NM_000399	EGR2	9,158	8,682	0,002	0,002	0,719	0,205	-1,392
F12	NM_000043	FAS	7,910	7,441	0,004	0,006	0,722	0,287	-1,385
G01	NM_002006	FGF2	3,386	2,331	0,096	0,199	0,481	0,105	-2,078
G02	NM_005860	FSTL3	8,542	8,225	0,003	0,003	0,803	0,546	-1,245
G03	NM_002229	JUNB	6,539	6,885	0,011	0,008	1,271	0,280	1,271
G04	NM_181712	KANK4	9,016	9,558	0,002	0,001	1,456	0,398	1,456
G05	NM_017644	KLHL24	3,952	4,983	0,065	0,032	2,044	0,049	2,044
G06	NM_002514	NOV	10,857	10,207	0,001	0,001	0,637	0,104	-1,569
G07	NM_020182	PMEPA1	4,910	4,602	0,033	0,041	0,808	0,410	-1,238
G08	NM_000602	SERPINE1	6,112	5,602	0,014	0,021	0,702	0,361	-1,424
G09	NM_005585	SMAD6	7,592	7,658	0,005	0,005	1,047	0,987	1,047
G10	NM_003107	SOX4	4,208	3,839	0,054	0,070	0,774	0,200	-1,292
G11	NM_015000	STK38L	3,976	3,686	0,064	0,078	0,818	0,116	-1,223
G12	NM_032873	UBASH3B	4,505	6,189	0,044	0,014	3,212	0,069	3,212
H01	NM_001101	ACTB	-4,107	-4,364	17,235	20,593	0,837	0,050	-1,195
H02	NM_004048	B2M	2,166	2,259	0,223	0,209	1,067	0,503	1,067
H03	NM_002046	GAPDH	-2,707	-2,589	6,530	6,015	1,086	0,376	1,086
H04	NM_000194	HPRT1	4,654	3,991	0,040	0,063	0,631	0,001	-1,584
H05	NM_001002	RPLP0	-4,113	-3,662	17,301	12,654	1,367	0,003	1,367

Listed here are the complete data from the Qiagen® PAHS-035YA array (Cat. No. 330231). The analysis includes n=3 independent mounts of iPSC ACVRL1$^{wt/wt}$ and iPSC ACVRL1$^{wt/mut}$, respectively. Regulation is expressed as change in expression of iPSC ACVRL1$^{wt/mut}$ compared with iPSC ACVRL1$^{wt/wt}$. Abbreviations of symbols in alphabetical order: ACTB: Actin, beta; ACTC1: Actin, alpha, cardiac muscle 1; ACVR: Activin A receptor (type I, IIA); AMH: Anti-Mullerian hormone; AMHR2: Anti-Mullerian hormone receptor, type II; ATF4: Activating transcription factor 4 (tax-responsive enhancer element B67); B2M: Beta-2-microglobulin; BAMBI: BMP and activin membrane-bound inhibitor homolog (Xenopus laevis;), BGLAP: Bone gamma-carboxyglutamate (gla) protein; BMP: Bone morphogenetic protein (1, 2, 3, 4, 5, 6, 7); BMPER: BMP binding endothelial regulator; BMPR: Bone morphogenetic protein receptor (type IA, IB, 2); CDKN: Cyclin-dependent kinase inhibitor (1A (p21, Cip1), 1B (p27, Kip1) 2B (p15, inhibits CDK4)); CHRD: Chordin; COL1A: Collagen, type I, alpha (1, 2); DCN: Decorin; EGR2: Early growth response 2; nEMP1: Epithelial membrane protein 1; ENG: Endoglin; FAS: Fas (TNF receptor superfamily, member 6); FGF2: Fibroblast growth factor 2 (basic); FSTL3: Follistatin-like 3 (secreted glycoprotein); GADD45B: Growth arrest and DNA-damage-inducible, beta; GDF: Growth differentiation factor (2, 3, 5, 6,7); GAPDH: Glyceraldehyde-3-phosphate dehydrogenase; GSC: Goosecoid homeobox; HPRT1: Hypoxanthine phosphoribosyltransferase 1; HERPUD1: Homocysteine-inducible, endoplasmic reticulum stress-inducible, ubiquitin-like domain member 1; ID1/2 : Inhibitor

of DNA binding 1/2 dominant negative helix-loop-helix protein; IFRD1: Interferon-related developmental regulator 1; IGF1: Insulin-like growth factor 1 (somatomedin C); IGFBP3: Insulin-like growth factor binding protein 3; IL6: Interleukin 6 (interferon, beta 2); INHA: Inhibin, alpha; JUNB: Jun B proto-oncogene; KANK4: KN motif and ankyrin repeat domains 4; KLHL24: Kelch-like 24 (Drosophila); LEFTY1: Left-right determination factor 1; LTBP1: Latent transforming growth factor beta binding protein; MYC V-myc: myelocytomatosis viral oncogene homolog (avian); NODAL: Nodal homolog (mouse); NOG: Noggin; NOV: Nephroblastoma overexpressed gene; PDGFB: Platelet-derived growth factor beta polypeptide; PLAU: Plasminogen activator, urokinase; PMEPA1: Prostate transmembrane protein, androgen induced 1; RPLP0: Ribosomal protein, large, P0; SMAD: SMAD family member (1, 2, 3, 4, 5, 7); SMURF1: specific E3 ubiquitin protein ligase 1; STAT1: Signal transducer and activator of transcription 1, 91kDa; TGFB1: Transforming growth factor, beta 1;TGFB1I1: Transforming growth factor beta 1 induced transcript 1; TGFB: Transforming growth factor, beta (2, 3); TGFBI: Transforming growth factor, beta-induced, 68kDa;TGFBR: Transforming growth factor, beta receptor (1, 2 (II (70/80kDa)), 3 (III); TGIF1: TGFB-induced factor homeobox 1; THBS1: Thrombospondin 1; TNFSF10: Tumor necrosis factor (ligand) superfamily, member 10; SERPINE1: Serpin peptidase inhibitor, clade E (nexin, plasminogen activator inhibitor type1), member 1; SMAD6: SMAD family member 6; SOX4: SRY (sex determining region Y)-box 4; STK38L: Serine/threonine kinase 38 like; UBASH3B: Ubiquitin associated and SH3 domain containing B

Table S2. Results of RT² Profiler PCR Array: Human TGFβ Signaling Targets (Qiagen® PAHS-235ZA)

Well	RefSeq	Symbol	AVG ΔCt		2^-ΔCt		fold change	*t*-test p	fold Up- or Down-Regulation
			ACVRL1-wt/mut	ACVRL1-wt/wt	ACVRL1-wt/mut	ACVRL1-wt/wt	ACVRL1-wt/mut to wt/wt	value	ACVRL1- wt/mut to wt/wt
A01	NM_001613	ACTA2	6,934	8,072	0,008	0,004	2,202	0,068	2,202
A02	NM_001105	ACVR1	5,571	5,032	0,021	0,031	0,689	0,024	-1,452
A03	NM_000020	ACVRL1	11,351	10,498	0,000	0,001	0,553	0,803	-1,807
A04	NM_000029	AGT	12,761	12,886	0,000	0,000	1,091	0,919	1,091
A05	NM_014336	AIPL1	11,071	10,954	0,000	0,001	0,922	0,595	-1,085
A06	NM_000044	AR	5,910	5,446	0,017	0,023	0,725	0,220	-1,380
A07	NM_001674	ATF3	3,997	5,685	0,063	0,019	3,221	0,006	3,221
A08	NM_001675	ATF4	-0,111	0,076	1,080	0,949	1,138	0,327	1,138
A09	NM_001186	BACH1	3,485	3,315	0,089	0,101	0,889	0,371	-1,125
A10	NM_138578	BCL2L1	5,426	4,227	0,023	0,053	0,436	0,002	-2,296
A11	NM_001709	BDNF	9,685	9,301	0,001	0,002	0,767	0,437	-1,304
A12	NM_003670	BHLHE40	3,968	5,238	0,064	0,027	2,410	0,074	2,410
B01	NM_005104	BRD2	3,517	3,116	0,087	0,115	0,758	0,034	-1,320
B02	NM_001254	CDC6	2,691	2,406	0,155	0,189	0,821	0,176	-1,218
B03	NM_004064	CDKN1B	4,867	4,723	0,034	0,038	0,905	0,520	-1,105
B04	NM_005194	CEBPB	6,058	5,041	0,015	0,030	0,494	0,001	-2,024
B05	NM_004379	CREB1	4,494	4,091	0,044	0,059	0,756	0,007	-1,322
B06	NM_004380	CREBBP	4,909	4,512	0,033	0,044	0,760	0,033	-1,316
B07	NM_001885	CRYAB	10,072	8,400	0,001	0,003	0,314	0,001	-3,187
B08	NM_001904	CTNNB1	2,563	2,134	0,169	0,228	0,743	0,332	-1,346
B09	NM_001539	DNAJA1	0,601	0,141	0,659	0,907	0,727	0,041	-1,376
B10	NM_001950	E2F4	4,017	3,238	0,062	0,106	0,583	0,001	-1,716
B11	NM_001423	EMP1	12,457	to l. v.	0,000	s. i.	no s. p.	no s. p.	no s. p.
B12	NM_000118	ENG	7,591	7,389	0,005	0,006	0,869	0,476	-1,150
C01	NM_001429	EP300	3,606	2,733	0,082	0,150	0,546	0,000	-1,833
C02	NM_004442	EPHB2	6,481	6,028	0,011	0,015	0,731	0,230	-1,369
C03	NM_002026	FN1	0,385	0,957	0,766	0,515	1,487	0,156	1,487
C04	NM_005252	FOS	4,246	4,369	0,053	0,048	1,088	0,598	1,088
C05	NM_002569	FURIN	4,825	4,430	0,035	0,046	0,760	0,015	-1,315
C06	NM_015675	GADD45B	3,253	4,054	0,105	0,060	1,742	0,025	1,742
C07	NM_005270	GLI2	7,800	6,574	0,004	0,010	0,428	0,005	-2,338
C08	NM_001518	GTF2I	0,350	0,178	0,784	0,884	0,888	0,316	-1,127
C09	NM_014685	HERPUD1	5,490	5,569	0,022	0,021	1,056	0,856	1,056
C10	NM_005524	HES1	3,345	3,951	0,098	0,065	1,522	0,317	1,522
C11	NM_012258	HEY1	6,706	6,929	0,010	0,008	1,168	0,803	1,168
C12	NM_002133	HMOX1	5,917	5,399	0,017	0,024	0,698	0,697	-1,432
D01	NM_002165	ID1	1,933	1,826	0,262	0,282	0,929	0,705	-1,077
D02	NM_002166	ID2	2,079	3,696	0,237	0,077	3,067	0,004	3,067
D03	NM_002167	ID3	1,307	1,512	0,404	0,351	1,153	0,373	1,153
D04	NM_001550	IFRD1	4,975	4,227	0,032	0,053	0,595	0,225	-1,680
D05	NM_000572	IL10	12,520	13,201	0,000	0,000	1,603	0,109	1,603
D06	NM_005655	KLF10	4,240	4,277	0,053	0,052	1,026	0,686	1,026
D07	NM_003188	MAP3K7	3,519	3,536	0,087	0,086	1,012	0,939	1,012
D08	NM_001315	MAPK14	4,433	3,861	0,046	0,069	0,673	0,069	-1,486
D09	NM_002750	MAPK8	4,015	3,867	0,062	0,069	0,903	0,298	-1,108
D10	NM_015844	MBD1	5,523	4,673	0,022	0,039	0,555	0,000	-1,803
D11	NM_004530	MMP2	4,117	3,707	0,058	0,077	0,753	0,027	-1,328
D12	NM_002449	MSX2	6,300	7,143	0,013	0,007	1,794	0,140	1,794
E01	NM_002467	MYC	1,833	1,963	0,281	0,256	1,095	0,482	1,095
E02	NM_002478	MYOD1	12,911	13,279	0,000	0,000	1,291	0,506	1,291
E03	NM_005596	NFIB	6,365	6,320	0,012	0,013	0,970	0,818	-1,031

E04	NM_020529	NFKBIA	3,609	3,975	0,082	0,064	1,289	0,265	1,289
E05	NM_017617	NOTCH1	4,768	4,241	0,037	0,053	0,694	0,014	-1,441
E06	NM_002607	PDGFA	0,857	1,042	0,552	0,486	1,137	0,509	1,137
E07	NM_000301	PLG	n. d.	13,302	absent	0,000	no s. p.	no s. p.	no s. p.
E08	NM_005036	PPARA	6,111	6,368	0,014	0,012	1,195	0,747	1,195
E09	NM_000963	PTGS2	11,019	10,718	0,000	0,001	0,811	0,210	-1,232
E10	NM_002820	PTHLH	12,639	11,325	0,000	0,000	0,402	0,168	-2,487
E11	NM_005607	PTK2	2,597	2,686	0,165	0,155	1,064	0,711	1,064
E12	NM_004103	PTK2B	9,307	9,493	0,002	0,001	1,138	0,544	1,138
F01	NM_006265	RAD21	1,706	1,506	0,306	0,352	0,870	0,131	-1,149
F02	NM_000964	RARA	5,408	4,774	0,024	0,037	0,644	0,037	-1,552
F03	NM_002895	RBL1	6,501	5,358	0,011	0,024	0,453	0,023	-2,208
F04	NM_001664	RHOA	0,824	1,222	0,565	0,429	1,318	0,037	1,318
F05	NM_004040	RHOB	4,015	3,869	0,062	0,068	0,904	0,612	-1,107
F06	NM_001754	RUNX1	9,024	8,912	0,002	0,002	0,925	0,819	-1,081
F07	NM_012234	RYBP	3,588	to l. v.	0,083	s. i.	no s. p.	no s. p.	no s. p.
F08	NM_002964	S100A8	to l. v.	n. d.	s. i.	absent	no s. p.	no s. p.	no s. p.
F09	NM_000602	SERPINE1	5,918	5,280	0,017	0,026	0,643	0,195	-1,556
F10	NM_000193	SHH	10,104	10,006	0,001	0,001	0,935	0,802	-1,070
F11	NM_005900	SMAD1	4,467	4,243	0,045	0,053	0,856	0,239	-1,168
F12	NM_005902	SMAD3	12,702	9,712	0,000	0,001	0,126	0,317	-7,940
G01	NM_005903	SMAD5	3,469	3,065	0,090	0,119	0,756	0,001	-1,323
G02	NM_005585	SMAD6	7,326	7,329	0,006	0,006	1,002	0,932	1,002
G03	NM_005985	SNAI1	7,793	7,097	0,005	0,007	0,617	0,025	-1,620
G04	NM_003107	SOX4	4,271	3,491	0,052	0,089	0,582	0,018	-1,717
G05	NM_138473	SP1	4,153	3,511	0,056	0,088	0,641	0,050	-1,560
G06	NM_004599	SREBF2	1,975	1,618	0,254	0,326	0,781	0,101	-1,280
G07	NM_003238	TGFB2	7,172	7,854	0,007	0,004	1,605	0,060	1,605
G08	NM_003242	TGFBR2	6,088	5,548	0,015	0,021	0,688	0,004	-1,454
G09	NM_003246	THBS1	4,133	3,357	0,057	0,098	0,584	0,140	-1,713
G10	NM_003810	TNFSF10	9,938	12,317	0,001	0,000	5,204	0,057	5,204
G11	NM_006472	TXNIP	4,158	5,807	0,056	0,018	3,136	0,083	3,136
G12	NM_003376	VEGFA	3,273	4,656	0,103	0,040	2,609	0,011	2,609
H01	NM_001101	ACTB	-4,215	-4,409	18,567	21,245	0,874	0,008	-1,144
H02	NM_004048	B2M	2,224	2,322	0,214	0,200	1,070	0,275	1,070
H03	NM_002046	GAPDH	-2,566	-2,633	5,921	6,202	0,955	0,682	-1,048
H04	NM_000194	HPRT1	4,651	4,080	0,040	0,059	0,673	0,000	-1,485
H05	NM_001002	RPLP0	-4,309	-3,769	19,824	13,635	1,454	0,004	1,454

Listed here are the complete data from the Qiagen® PAHS-235ZA (Cat. No. 330231). The analysis includes n=3 independent mounts of iPSC ACVRL1$^{wt/wt}$ and iPSC ACVRL1$^{wt/mut}$, respectively. Regulation is expressed as change in expression of iPSC ACVRL1$^{wt/mut}$ compared with iPSC ACVRL1$^{wt/wt}$. Index of abbreviations within the measurement values: n. d.: not detectable; no s.p.: no statement possible; s. i.: sometimes induced; to l. v.: to less values. Abbreviations of symbols in alphabetical order: ACTA2: Actin, alpha 2, smooth muscle, aorta; ACTB: Actin, beta; ACVR1: Activin A receptor, type I; ACVRL1: Activin A receptor type II-like 1; AGT: Angiotensinogen (serpin peptidase inhibitor, clade A, member 8); AIPL1: Aryl hydrocarbon receptor interacting protein-like 1; AR: Androgen receptor; ATF: Activating transcription factor (3, 4 (tax-responsive enhancer element B67)); B2M: Beta-2-microglobulin; BACH1: BTB and CNC homology 1, basic leucine zipper transcription factor 1; BCL2L1: BCL2L1 BCL2-like 1; BDNF: Brain-derived neurotrophic factor; BHLHE40: Basic helix-loop-helix family, member e40; BRD2: Bromodomain containing 2; CDC6: Cell division cycle 6 homolog (S. cerevisiae); CDKN1B: Cyclin-dependent kinase inhibitor 1B (p27, Kip1); CEBPB: CCAAT/enhancer binding protein (C/EBP), beta; CREB1: CAMP responsive element binding protein 1; CREBB:P CREB binding protein; CRYAB: Crystallin, alpha B; CTNNB1: Catenin (cadherin-associated protein), beta 1, 88kDa; DNAJA1: DnaJ (Hsp40) homolog, subfamily A, member 1; E2F4: E2F transcription factor 4, p107/p130-binding; EMP1:

Epithelial membrane protein 1; ENG: Endoglin; EP300: E1A binding protein p300; EPHB2: EPH receptor B2; FN1: Fibronectin 1; FOS: FBJ murine osteosarcoma viral oncogene homolog; FURIN: Furin (paired basic amino acid cleaving enzyme); GADD45B: Growth arrest and DNA-damage-inducible, beta; GAPDH: Glyceraldehyde-3-phosphate dehydrogenase; GLI2: GLI family zinc finger 2; GTF2I: General transcription factor Iii; HERPUD1: Homocysteine-inducible, endoplasmic reticulum stress-inducible, ubiquitin-like domain member 1; HES1: Hairy and enhancer of split 1, (Drosophila); HEY1: Hairy/enhancer-of-split related with YRPW motif 1; HMOX1: Heme oxygenase (decycling) 1; HPRT1: Hypoxanthine phosphoribosyltransferase 1; ID: Inhibitor of DNA binding (1, 2, 3) dominant negative helix-loop-helix protein; IFRD1: Interferon-related developmental regulator 1; IL10: IL10 Interleukin 10; KLF10: Kruppel-like factor 10; MAP3K7: Mitogen-activated protein kinase kinase kinase 7; MAPK: Mitogen-activated protein kinase (14, 8); MBD1: Methyl-CpG binding domain protein 1 MMP2: Matrix metallopeptidase 2 (gelatinase A, 72kDa gelatinase, 72kDa type IV collagenase); MSX2: Msh homeobox 2; MYC: V-myc myelocytomatosis viral oncogene homolog (avian); MYOD1: Myogenic differentiation 1; NFIB: Nuclear factor I/B; NFKBIA: Nuclear factor of kappa light polypeptide gene enhancer in B-cells inhibitor, alpha; NOTCH1: Notch 1; PDGFA: Platelet-derived growth factor alpha polypeptide; PLG: Plasminogen; PPARA: Peroxisome proliferator-activated receptor alpha; PTGS2: Prostaglandin-endoperoxide synthase 2 (prostaglandin G/H synthase and cyclooxygenase); PTHLH: Parathyroid hormone-like hormone; PTK2: PTK2 protein tyrosine kinase 2; PTK2B: PTK2B protein tyrosine kinase 2 beta; RAD21 RAD21 homolog (S. pombe); RARA: Retinoic acid receptor, alpha; RBL1: Retinoblastoma-like 1 (p107); RHO: Ras homolog gene family, (A (member A), B (member B)); RPLP0: Ribosomal protein, large, P0; RUNX1: Runt-related transcription factor 1; RYBP: RING1 and YY1 binding protein; S100A8: S100 calcium binding protein A8; SERPINE1. Serpin peptidase inhibitor, clade E (nexin, plasminogen activator inhibitor type 1), member 1; SHH: Sonic hedgehog; SMAD: SMAD family member (1,3 5, 6); SNAI1: Snail homolog 1 (Drosophila); SOX4: SRY (sex determining region Y)-box 4; SP1: Sp1 transcription factor; SREBF2: Sterol regulatory element binding transcription factor 2; TGFB2: Transforming growth factor, beta 2; TGFBR2: Transforming growth factor, beta receptor II (70/80kDa); THBS1: Thrombospondin 1; TNFSF10: Tumor necrosis factor (ligand) superfamily, member 10; TXNIP: Thioredoxin interacting protein; VEGFA: Vascular endothelial growth factor A

ALK1

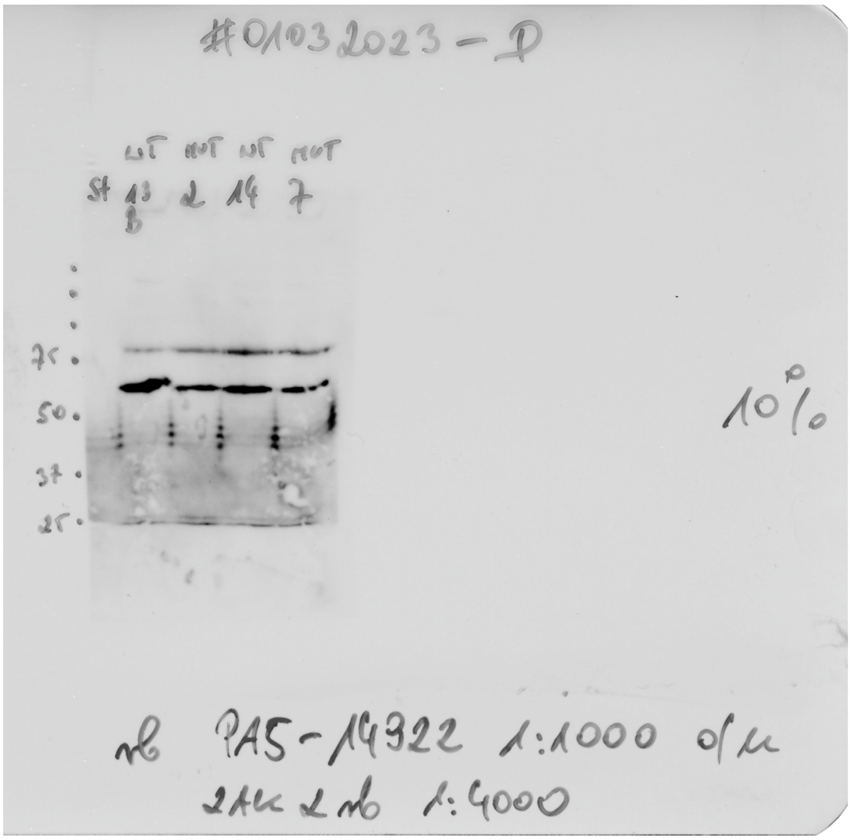
#01032023-D
WT MUT WT MUT
St 13 B 2 14 7
75
50
37
25
10%
Ab PA5-14322 1:1000 O/N
2Ak 2Ab 1:4000

GAPDH

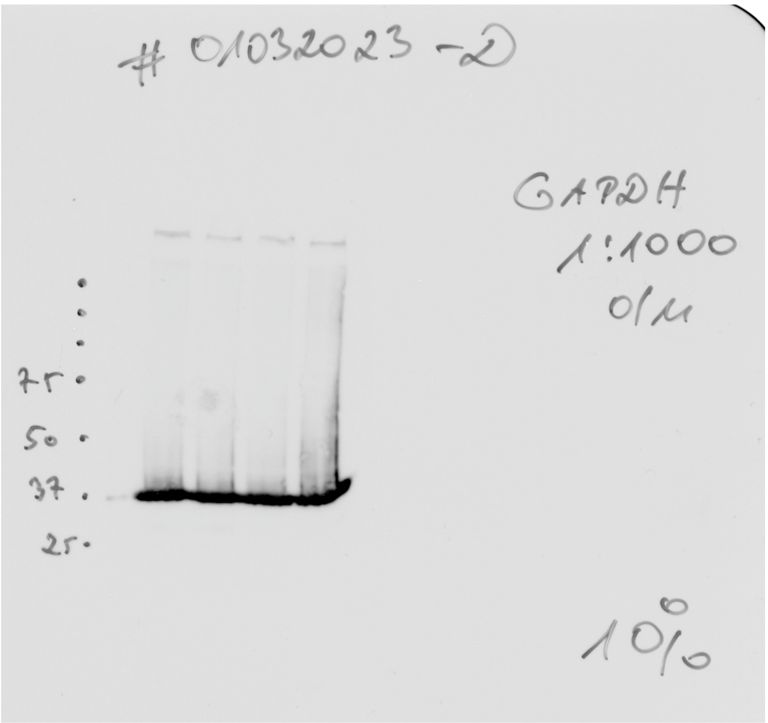
#01032023-D
GAPDH
1:1000
O/N
75
50
37
25
10%

TGF-beta 1

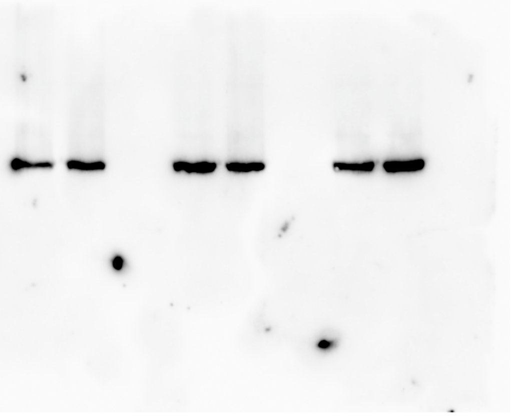

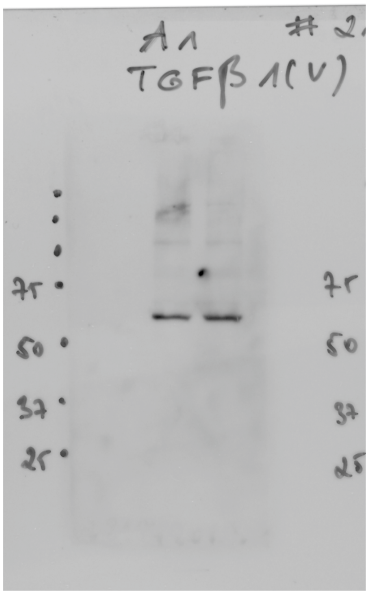

GAPDH

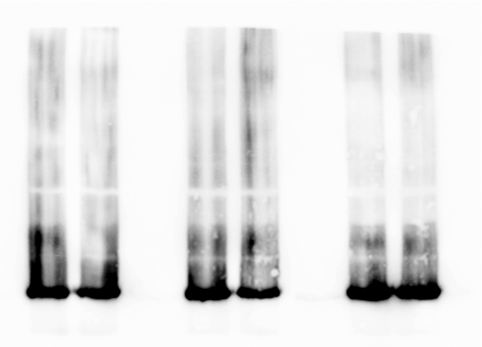

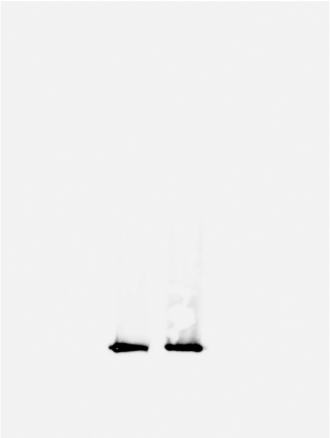

SMAD4

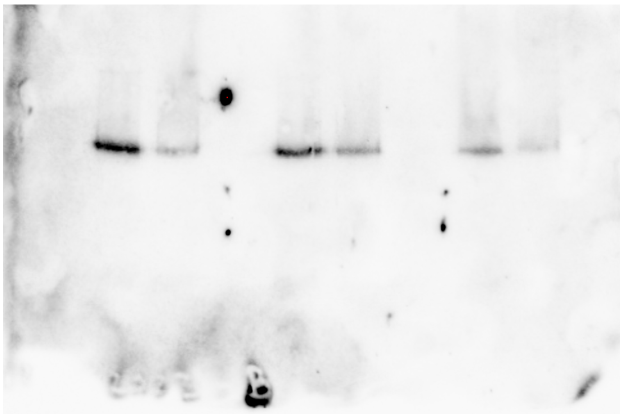

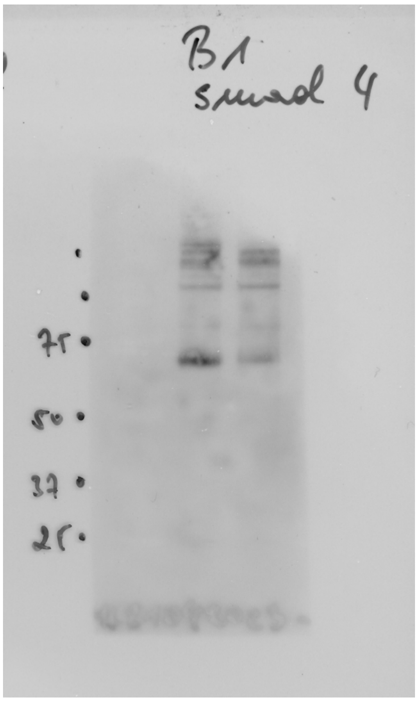

GAPDH

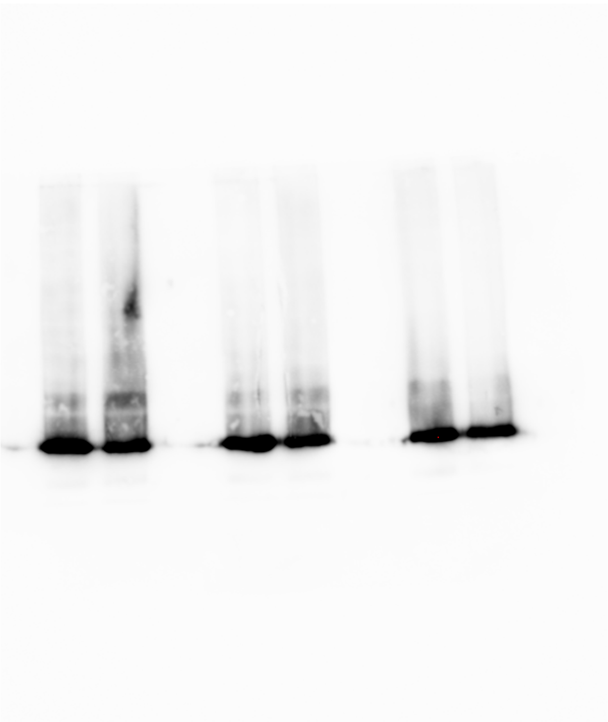

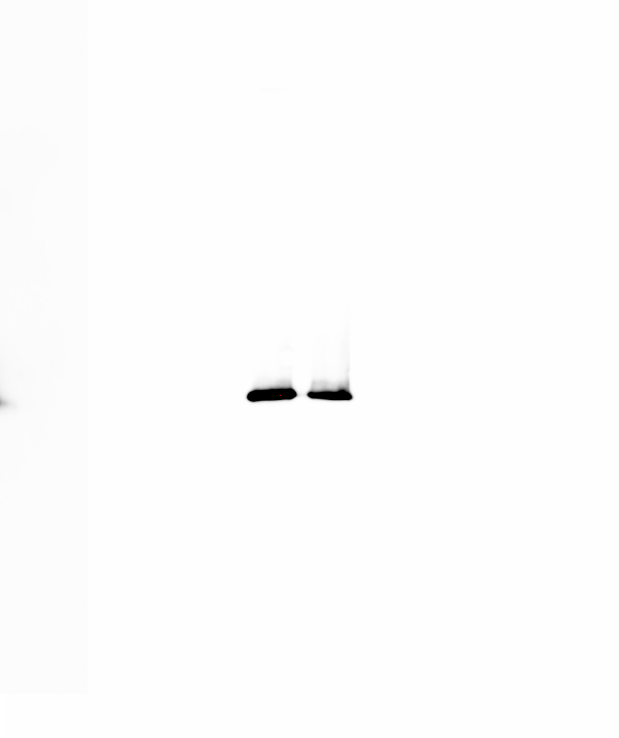

Supplement: Supplementary file 1 [file cells-12-01600-s001.zip › Supplementary files.pdf]
